# The Possibilities of Anti-Typhoid Serum

**Published:** 1906-04-28

**Authors:** 


### The Hospital

A Journal of

The Medical Sciences and Hospital Administration.

Vol. XL.—No. 1,022. SATURDAY, APRIL 28, 1906.

### The Possibilities of Anti-Typhoid Serum.

The treatment of typhoid fever is a question of ever-enduring interest to members of the medical profession, as it is also one of great practical moment to individual patients and to the general community. The disease is—or perhaps we should rather say until recent days was—so frequent and so widely distributed that its practical demands in the shape of treatment are familiar to the majority of medical men, and, indeed, many a general practitioner has on this point a more abundant basis of experience than is possessed by the average consulting physician. Hence the subject is the reverse of academic, and it makes its appeal both to those immediately responsible for the individual patient and to those concerned with the care of the public health. It would be idle to pretend that this general distribution of the disease has resulted in a unanimous opinion in reference to all the details of treatment. Yet it may be fairly stated that whilst special virtues have been claimed for particular agents and methods so far as the diminution of risks and the avoidance of complications are concerned, there has been no considerable dissent from the proposal that no actual specific for the disease is included within the therapeutist's armamentarium.

But when investigations into the bacteriology of various diseases and into the nature of infection and the causes of immunity produced an appreciation of the possibilities of serums and vaccines as prophylactic and curative agents, it was natural to anticipate that a new chapter had been opened in the therapeutics of the specific fevers. How successfully that anticipation has been realised in the case of diphtheria is now a matter of common knowledge. In other diseases, however, a much less secure footing has been attained, and practical results have been both less frequent and less conspicuous. It is in this latter category that, so far as specific treatment is concerned, typhoid fever must still be placed. In addition, however, to a healthy optimism, based upon confidence in the progress of scientific investigation and research, there are definite grounds for expecting that this position may be largely and beneficially modified. It has been shown, for example, that various animals may be rendered immune to the typhoid virus by the use of dead cultures as injections, and, as is now well known, Professor A. E. Wright claims that by the use of a properly prepared vaccine human beings can be protected against the disease, or at least against its more serious developments. When, however, the reports of the treatment by "anti-typhoid" serums of patients actually suffering from typhoid fever are studied, there does not appear so much ground for encouragement. These serums are for the most part prepared by immunising horses with the *Bacillus typhosus*, and are bactericidal and not anti-toxic in action. Of them a competent authority writes: "They have given no marked assistance in the treatment of the disease in man." There is, however, another aspect of the matter, and this is provided by the work of Chantemesse. As a physician for contagious diseases in one of the "bastions" of Paris, Chantemesse has had under his care, during the last five years, 712 cases of typhoid fever, and has treated these with a special anti-typhoid serum. He reports a total of 27 deaths—that is, a mortality of 3.7 per cent., as compared with a mortality of 17.3 per cent. occurring among the 3,595 cases of the disease treated during the same period of time in the other hospitals of the city. The serum is prepared by inoculating horses with the toxins of the typhoid bacillus. In action it is anti-toxic and thus causes a rapid disappearance of the diarrhœa, headache, and other symptoms of general toxæmia. The suggestion is that the efficacy of the serum is due to its power of increasing the defensive power of the body against the typhoid toxins. In favour of this it is shown, first, that the use of the serum is followed by changes in the blood identical with those which take place spontaneously during normal convalescence; and, secondly, that at the same time there is a temporary enlargement of the spleen and probably also of other collections of lymphoid tissue, in which, as it may be said, resistance to the typhoid invasion is normally organised. These facts, and more especially the last mentioned, emphasise the importance of early diagnosis and prompt injection, for it is obvious that when the lymphoid tissues have for a relatively long period been prejudiced by the specific toxins their ability to respond to the call of the serum must be correspondingly diminished. In addition to a great reduction of case-mortality Chantemesse claims that his treatment considerably shortens the duration of the disease and avoids the risk of serious complications. Thus, whilst in his 712 cases there were 9 deaths due to perforation, this complication never occurred in any case treated by the serum within seven days after the appearance of the fever. Here, again, emphasis is lent to the demand for early diagnosis, and this is, as everyone knows, one of the difficulties of the situation. Widal's reaction has done much, but a still earlier criterion is required. Inquiry is being turned in this direction, and if, or rather when, it proves successful, Chantemesse's work encourages the hope that the specific treatment of typhoid fever is soon to be added to the triumphs of practical therapeutics.

